# Reactive Compatibilization of Polyamide 6/Olefin Block Copolymer Blends: Phase Morphology, Rheological Behavior, Thermal Behavior, and Mechanical Properties

**DOI:** 10.3390/ma13051146

**Published:** 2020-03-05

**Authors:** Xintu Lin, Yuejun Liu, Xi Chen, Yincai Wu, Lingna Cui, Long Mao, Wei Zheng, Minghao Lin

**Affiliations:** 1 Key Laboratory of Advanced Packaging Materials and Technology of Hunan Province, School of Packaging and Materials Engineering, Hunan University of Technology, Zhuzhou 412007, China; xintulin@163.com (X.L.);; 2 Xiamen Changsu Industrial Company, Limited, Xiamen 361026, China; 3 Key Laboratory of Polymer Processing Principle and Application, Xiamen University of Technology, Xiamen 361024, China

**Keywords:** compatibilization, melt blending, olefin block copolymer, polyamide 6, rheology

## Abstract

In this study, the morphology, rheological behavior, thermal behavior, and mechanical properties of a polyamide 6 (PA6) and olefin block copolymer (OBC) blend compatibilized with maleic anhydride-grafted polyethylene-octene copolymer (POE-g-MAH) were investigated. The morphological observations showed that the addition of POE-g-MAH enhanced the OBC particle dispersion in the PA6 matrix, suggesting a better interfacial compatibility between the pure PA6 and OBC. The results of the Fourier transform infrared (FTIR) spectroscopy analysis and the Molau test confirmed the compatibilization reactions between POE-g-MAH and PA6. The rheological test revealed that the melt viscosity, storage modulus (G’), and loss modulus (G”) of the compatibilized PA6/OBC blends at low frequency were increased with the increasing POE-g-MAH content. The thermal analysis indicated that the addition of OBC had little effect on the crystallization behavior of PA6, while the incorporation of POE-g-MAH at high content (7 wt%) in the PA6/OBC blend restricted the crystallization of PA6. In addition, the compatibilized blends exhibited a significant enhancement in impact strength compared to the uncompatibilized PA6/OBC blend, in which the highest value of impact strength obtained at a POE-g-MAH content of 7 wt% was about 194% higher than that of pure PA6 under our experimental conditions.

## 1. Introduction

Polymer blending modification is a widely used method to obtain materials with improved properties [1,2,3]. Many polymers, including poly(ethylene terephthalate) (PET) [4], polypropylene (PP) [5], poly(butylene terephthalate) (PBT) [6], polyamide [7], and poly(lactic acid) (PLA) [8,9], have been modified by blending modification.

Polyamide 6 (PA6), a typical semi-crystalline polymer with excellent chemical and physical properties, has been widely used in automotive industries, electronics, chemical industries, and films, etc. However, the notched impact strength of PA6 is low, especially at low temperatures, which limits its wide applications. As a result, many efforts have been devoted to improving the toughness of PA6 by melt blending with flexible polymers and elastomers, such as low density polyethylene (LDPE) [10], high density polyethylene (HDPE) [11,12], acrylonitrile-butadiene-styrene (ABS) [13,14], poly(ethylene-octene) (POE) [15,16], ethylene-propylene-diene rubber (EPDM) [17], ethylene vinyl acetate (EVA) [18], maleated styrene-ethylene-butylene-styrene (SEBS-g-MAH) [19], and maleated ethylene-octene copolymer (EOC-g-MAH) [20].

However, PA6 and flexible polymers and elastomers are often immiscible due to the difference of polarities, leading to a phase-separated morphology and poor compatibility between the PA6 matrix and the dispersed phase, which will result in unsatisfactory mechanical properties. Therefore, improving the compatibility between PA6 and flexible polymers and elastomers is critically important to achieve a high fracture toughness of the blends. Generally, blending PA6 with functionalized flexible polymers or functionalized elastomers or adding a third component, like a copolymer or compatibilizer, is a widely used method to improve the compatibility between PA6 and flexible polymers and elastomers.

The functional group of the modified flexible polymer and compatibilizer, which is usually maleic anhydride or an epoxy group, can react with the amine groups of PA6 to form a graft copolymer, which can improve the compatibility between the PA6 matrix and the dispersed phase and then obtain the desired properties of the blends [21,22,23,24]. Xie et al. [15] prepared a series of POE-g-MAH as the compatibilizer for a PA6/POE blend, and they found that the increase of the grafting degree of maleic anhydride in the POE-g-MAH increased the notched Izod impact strength of the compatibilized PA6/POE blend. Das et al. [25] revealed that the mechanical properties of PA6/linear low-density polyethylene (PA6/LLDPE) blends were increased by using maleated LLDPE (LLDPE-g-MAH) as the reactive compatibilizer. Huang et al. [26] found that the impact strength of a PA6/ABS blend containing 20 wt% ethylene-acrylate-glycidyl methacrylate copolymer (EAGMA) was about 323% higher than that of pure PA6.

Recently, olefin block copolymer (OBC), a novel thermoplastic polyolefin elastomer, was synthesized by the Dow Chemical Company through the method of chain shuttling polymerization [27]. Compared with random ethylene-octene copolymers, OBC exhibits a higher melting temperature, higher heat resistance and lower glass transition temperature, while maintaining excellent elastomeric properties at high temperatures [28]. The high toughness of OBC makes it a excellent toughening agent for plastics, and many studies have demonstrated that OBC can be used as an effective toughening agent [29,30,31]. Ren et al. [29] found that the addition of OBC effectively increased the Izod impact strength of polypropylene random copolymers (PPR).

To the best of our knowledge, relatively few studies have been performed on the use of OBC as an impact modifier for PA6 based blends. Due to polarity difference between PA6 and OBC, it is necessary to improve the compatibility between the OBC dispersed phase and PA6 matrix to obtain the desired high mechanical performance. Previous researchers have reported that maleic anhydride-grafted POE (POE-g-MAH) can be used as an effective compatibilizing agent for PA6 based blends [15,32]. Thus, it was expected that the compatibility of PA6/OBC blends could be enhanced by using POE-g-MAH as a compatibilizer whose compatibilizing effects on this blend are not yet systematically investigated and reported in the literature.

In this work, the influence of a POE-g-MAH compatibilizer on the morphology, rheological behavior, thermal behavior, and mechanical properties of PA6/OBC blends was determined by using scanning electron microscopy (SEM), rheometry, differential scanning calorimetry (DSC) and tensile, flexural, and notched Charpy impact tests, respectively. Furthermore, Fourier transform infrared (FTIR) spectroscopy analysis and the Molau test were also carried out to explore the possible chemical interactions between the PA6 matrix and POE-g-MAH.

## 2. Experimental

### 2.1. Materials

Polyamide 6 (PA6) (Novamid^®^ 1010C2) with a density of 1.13 g/cm^3^ was purchased from the DSM Engineering Plastics, the Netherlands. Olefin block copolymer (OBC) (INFUSE ™ 9107) was supplied by the Dow Chemical Company, Michigan, USA. The characteristics of the OBC are as follows: a density of 0.866 g/cm^3^, a melt index of 1.0 g/10 min (190 °C, 2.16 Kg), ultimate tensile strength of 5.1 Mpa, and ultimate tensile elongation of 600%. Maleic anhydride-grafted poly(ethylene-octene) (POE-g-MAH, CMG5805-L) with 0.8 wt% maleic anhydride was obtained from Fine-blend Compatilizer Jiangsu Co., Ltd., Nantong, China. Formic acid (AR, 98%) was supplied by Sinopharm Chemical Reagent Co., Ltd, Shanghai, China.

### 2.2. Preparation of the Blends

PA6 pellets were dried at 80 °C for 12 h in a hot air oven. OBC and POE-g-MAH were dried using a vacuum oven at 50 °C for 12 h. Then, all specimens were prepared in a co-rotating twin-screw extruder (CHT35/600-18.5-40, L/D = 40, Nanjing Ruiya Polymer Processing Equipment Company, Nanjing, China). The screw speed was set at 120 rpm and the temperature profile from the feed zone to die zone was set at 215, 220, 225, 230, 230, 230, 235, 240, 240, and 235 °C, respectively. The extrudates were then chopped into small pellets. Finally, the obtained pellets were dried at 85 °C for 12 h and injection-molded into standard specimens using an injection molding machine (PL860-260, Haitian plastics machinery Co., Ltd., Wuxi, Jiangsu, China). The barrel temperatures were 210–240 °C, and the mold temperature was kept at 25 °C. The compositions of the PA6/OBC/POE-g-MAH blends under investigation are given in Table 1.

### 2.3. Characterization

Fourier transform infrared (FTIR) spectrometry (Nicolet iS5, Thermo Scientific, Madison, WI, USA) was used to study the possible chemical interactions between PA6 and POE-g-MAH.

The Molau test was performed by mixing about 103 mg of the samples in 20 mL of 98% formic acid in different test tubes. The mixture was stored at room temperature for 22 h.

The morphology of the blends was investigated using scanning electron microscopy (SEM) (MIRA3 LMU, TESCAN, Brno, Czech). The specimens were fractured in liquid nitrogen and then coated with gold. The number average diameter (D_n_), weight average diameter (D_w_), and polydispersity index (PDI) of the OBC particles were calculated using the following equations [33]:

(1) Dn=∑NDii∑Ni

(2) Dw=∑NDi2i∑NDii

(3) $$PDI=\frac{D_{w}}{D_{n}}$$

where $D_{i}$ is the diameter of OBC particles with the number $N_{i}$.

Rheological behaviors of the samples were measured using a rheometer (Discovery HR-2, TA Instruments, New Castle, DE, USA) with a plate diameter of 25 mm at 230 °C. The frequency sweep test was performed from 0.1 and 628 rad/s, and the strain was 0.5% under linear viscoelastic conditions.

Differential scanning calorimetry (DSC) (DSC1/500, Mettler Toledo, Zurich, Switzerland) was used to investigate the thermal properties of the samples. The specimens were initially heated from 30 °C to 260 °C at 20 °C /min and held at 260 °C for 5 min to erase the prior thermal history, and then cooled to 30 °C at 20 °C /min to determine the crystallization temperature (T_c_), and reheated again to 260 °C with the same heating rate to determine the melting temperature (T_m_). The degree of crystallinity (X_c_) of PA6 and OBC was calculated from the melting enthalpy values using the following Equation (4):

(4) $$X_{c}(\%)=\frac{\Delta H_{m}}{w_{f}\Delta H_{m}^{0}}*100$$

where $\Delta H_{m}$ is the melting enthalpy of PA6 or OBC in the samples, $w_{f}$ is the weight percent of PA6 or OBC in the samples, and $\Delta H_{m}^{0}$ is the melting enthalpy of 100% crystalline for PA6 (230 J/g) [34] and OBC (290 J/g) [35]).

**Figure 1:**
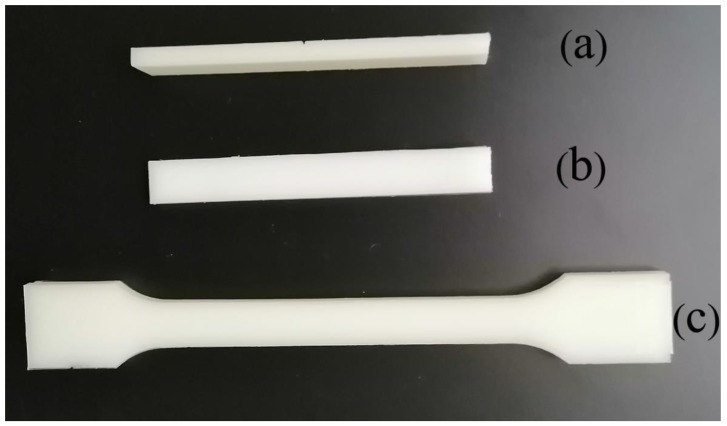
(**a**) Notched Charpy impact test specimen, (**b**) flexural test specimen, and (**c**) tensile test specimen.

Notched Charpy impact tests were performed on an impact tester (PIT501J, Shenzhen Wance Testing Machine Co., Ltd., Shenzhen, China) at room temperature according to Chinese Standard GB/T1043-1993, and the specimens (80 mm in length, 10 mm in width, and 4 mm in thickness) were used with a 45° V-shaped notch and a notch depth of 0.8 mm (Figure 1a). For each condition, more than five samples were tested to determine the average values.

Tensile and flexural tests were carried out using a micro-controlled electronic universal testing machine (ETM502B-Ex, Shenzhen Wance Testing Machine Co., Ltd., Shenzhen, China) at room temperature according to Chinese Standard GB/T1040-2006 and GB/T9341-2008, and crosshead speeds of 50 mm/min and 20 mm/min were used, respectively. The dimensions of the specimens for the flexural tests were 80 mm × 10 mm × 4 mm (length × width × thickness) (Figure 1b). The specimens for the tensile tests were dumbbell-shaped with dimensions of 150 mm × 10 mm × 4 mm (length × width narrow parallel portion × thickness) (Figure 1c). For each condition, five samples were tested, and the results were averaged arithmetically.

## 3. Results and Discussion

### 3.1. Fourier Transform Infrared (FTIR) Spectroscopy Analysis

**Figure 2:**
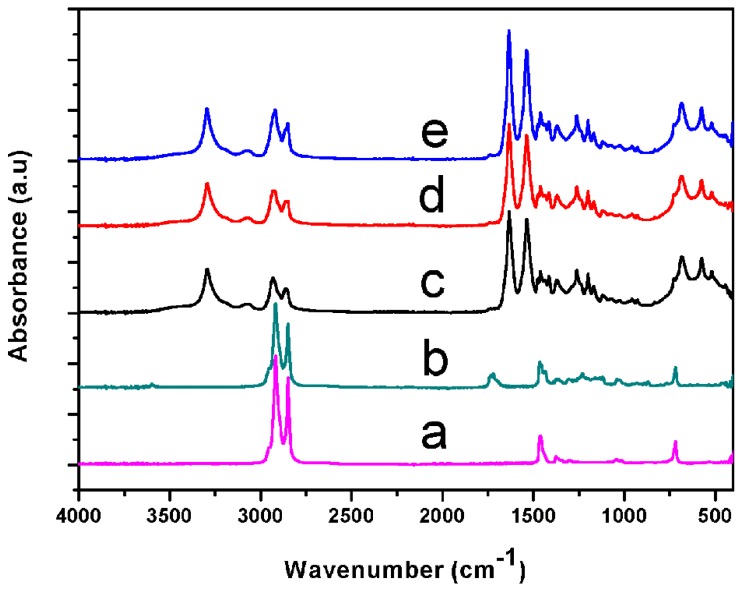
Fourier transform infrared (FTIR) spectra of (**a**) pure OBC, (**b**) pure POE-g-MAH, (**c**) pure PA6, (**d**) the uncompatibilized PA6/OBC blend, and (**e**) the 7 wt% POE-g-MAH compatibilized PA6/OBC blend (PA6/OBC/7).

Figure 2 depicts the FTIR spectra of pure PA6, pure OBC, and the PA6/OBC blends with and without POE-g-MAH. As shown in Figure 2, pure PA6 exhibited absorption characteristic bands at 1538 cm^−1^ (the combination of bending vibration of the N–H group and stretching vibration of the C–N group (Amide II, N–H bending + C–N stretching)), at 1633 cm^−1^ (stretching vibration of the C=O group (Amide I, C=O stretching)), at 2932 cm^−1^ (stretching vibration of the C–H group (C–H stretching)), as well as bands at 3294 cm^−1^ (stretching vibration of the N–H group (N–H stretching)). After addition of OBC and POE-g-MAH to PA6, the spectra of the resulting PA6/OBC and PA6/OBC/POE-g-MAH blends showed similar characteristic bands as those of PA6, and there was no appearance of new absorption bands.

**Figure 3:**
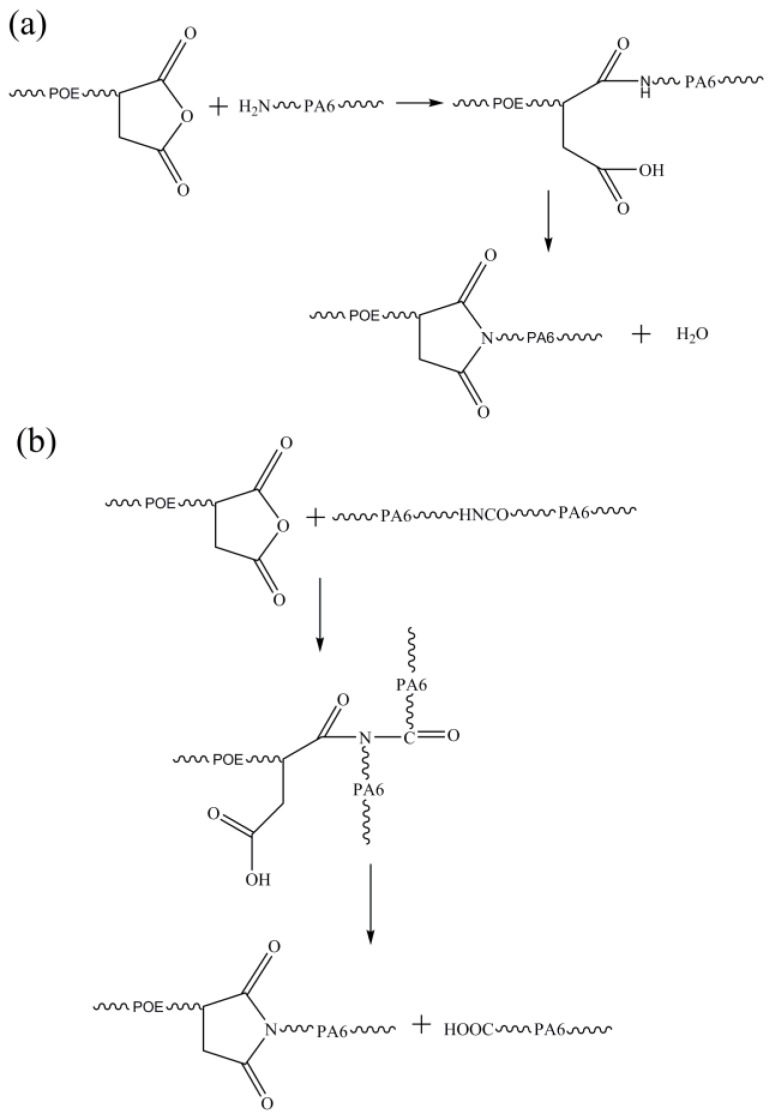
Possible chemical reaction between PA6 and POE-g-MAH. (**a**) the reaction between MAH groups of POE-g-MAH and –NH_2_ groups of PA6; (**b**) the reaction between MAH groups of POE-g-MAH and –NH– groups of PA6.

However, we noted that the N–H and C=O characteristic bands of PA6 identified on the spectra of the compatibilized PA6/OBC blend were slightly stronger than those of pristine PA6 and the umcompatibilized samples. In addition, the absorption characteristic bands corresponding to the maleic anhydride groups of POE-g-MAH disappeared in the compatibilized PA6/OBC blend. This observation indicates the occurrence of a reaction between the PA6 and the POE-g-MAH compatibilizer. As shown in Figure 3, when POE-g-MAH was incorporated into the uncompatibilized PA6/OBC blend, the amine groups of PA6 reacted with the maleic anhydride groups of POE-g-MAH to form the functional groups of N–H and C=O, which are overlapped with the original absorption characteristic bands of PA6. A similar observation was reported by Marco et al. [36] for the polypropylene functionalized with maleic anhydride (PP-g-MA) compatibilized isotactic polypropylene (iPP)/nylon 6 (PA6) blends. This finding is in correspondence with the result derived from the Molau test in the next section.

### 3.2. Molau Test

**Figure 4:**
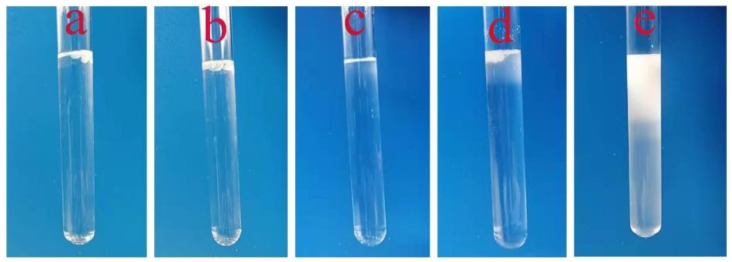
The Molau tests of (**a**) pure OBC, (**b**) pure POE-g-MAH, (**c**) pure PA6, (**d**) the uncompatibilized PA6/OBC blend, and (**e**) the 7 wt% POE-g-MAH compatibilized PA6/OBC blend (PA6/OBC/7).

The Molau test was used to confirm the formation of a grafted copolymer in the compatibilized PA6/OBC blend, and the results of Molau test of pure PA6, pure OBC, pure POE-g-MAH, the uncompatibilized PA6/OBC blend, and the 7 wt% POE-g-MAH compatibilized PA6/OBC blend (PA6/OBC/7) are shown in Figure 4. It was observed that pure PA6 was completely dissolved in formic acid and exhibited a transparent solution (Figure 4c), while pure OBC and pure POE-g-MAH was insoluble (Figure 4a,b). As shown in Figure 4d, for the uncompatibilized PA6/OBC blend, PA6 phase was dissolved in formic acid whereas the OBC phase floated on the top of the solution, indicating poor interfacial adhesion between the PA6 matrix and the OBC dispersed phase. However, a milky white solution was observed after the addition of 7 wt% POE-g-MAH to the PA6/OBC blend (Figure 4e). This phenomenon indicates that a grafted copolymer was formed between the PA6 and OBC phase during the melt blending, which can act as an emulsifying agent, and enhance the compatibility between the two phases.

### 3.3. Morphological Analyses

**Figure 5:**
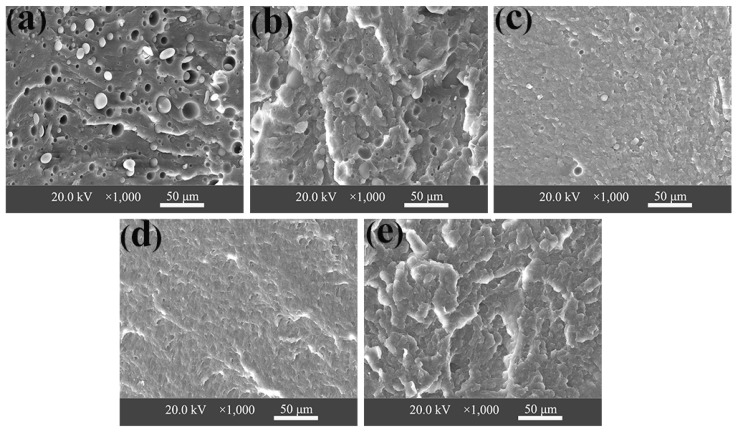
Scanning electron microscopy (SEM) images of the cryo-fractured surfaces of (**a**) PA6/OBC, (**b**) PA6/OBC/1, (**c**) PA6/OBC/3, (**d**) PA6/OBC/5 and (**e**) PA6/OBC/7.

The phase morphologies of the uncompatibilized and compatibilized PA6/OBC blends were investigated by SEM. Figure 5 delineates the cryo-fractured surfaces of PA6/OBC and the PA6/OBC/POE-g-MAH blends. For the uncompatibilized PA6/OBC blend (Figure 5a), the large voids with clear contours representing the OBC dispersed particles pulled out from the PA6 matrix, are readily observable on the fractured surfaces. The OBC particles were poorly dispersed in the PA6 matrix with a larger size of about 8.2 μm. This revealed the poor compatibility and weak interfacial interactions between the PA6 and the OBC phases. In general, the final morphologies of the immiscible blends are believed to be affected by the droplet break-up and coalescence [37,38]. According to the work of Sundararaj et al. [39], for uncompatibilized blends, higher concentrations of the dispersed phase caused larger particle sizes due to increased coalescence. With the addition of OBC, the coalescence of OBC particles became relatively obvious due to the high interfacial tension between the PA6 matrix and the OBC dispersed phase, resulting thus in coarsened morphology. The poor compatibility between the PA6 and OBC phase was also responsible for the limited improvement in the mechanical properties. Therefore, a suitable compatibilizer was needed to promote the dispersion of the OBC particles in the PA6 matrix.

When the POE-g-MAH was added to the PA6/OBC blend, the scale and the number of voids corresponding to the pulled out OBC particles decreased, and the particle size of the OBC phase decreased significantly with increasing POE-g-MAH content, as can be observed in Figure 5b,c. The average particle size of OBC in the uncompatibilized PA6/OBC blend was 8.2 μm (Figure 5a), whereas it decreased from 8.2 to 5.7 μm with the increase of POE-g-MAH contents from 0 to 3 wt% (Figure 5a,c). In addition, the PDI decreased from 1.29 for uncompatibilized PA6/OBC blend (Figure 5a) to 1.15 for PA6/OBC blends containing 3 wt% POE-g-MAH (Figure 5c). Hemelrijck et al. [40,41] suggested that the presence of compatibilizers could improve the compatibility between the phases, and resulted thus in a finer morphology by reducing the interfacial tension and suppressing droplet coalescence.

The decrease in particle size distribution and the average particle size of the OBC phase was probably due to the inhibition of the coalescence of the dispersed phase and the decrease of interfacial tension caused by the compatibilizing effect of POE-g-MAH. For the compatibilized PA6/OBC blends containing high concentrations of POE-g-MAH (>3 wt%), it was found that the OBC particles were almost invisible and the phase interface between the PA6 matrix and OBC phase became blurred (Figure 5d,e). This observation was indicative of the suppression of droplet coalescence and the improved compatibility and interface combination between the PA6 matrix and OBC phase. Therefore, the compatibilized PA6/OBC blends exhibited finer morphology of the dispersed phase, which was responsible of the increased mechanical properties of the PA6/OBC blends as discussed in the corresponding section. The results obtained from the morphological analyses are also consistent with those obtained from the rheological analyses discussed in the next section.

### 3.4. Rheological Behavior

**Figure 6:**
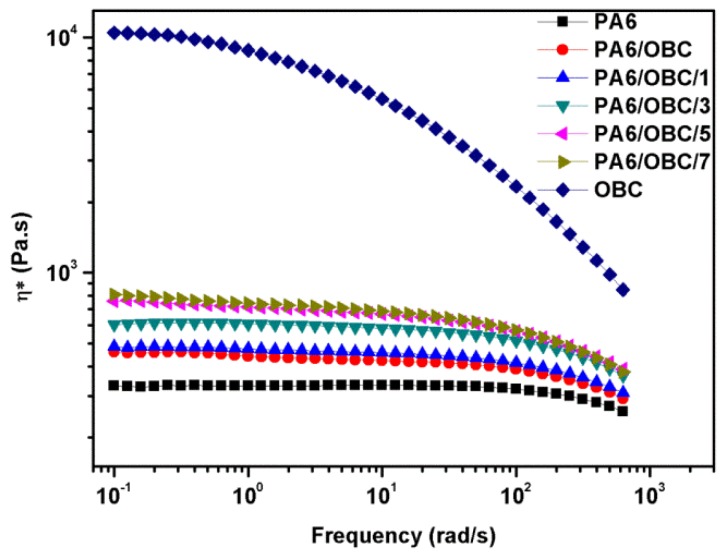
The complex viscosity of pure PA6, pure OBC, and the PA6/OBC and PA6/OBC/POE-g-MAH blends at various POE-g-MAH contents.

Figure 6 illustrates the complex viscosity (η*) of pure PA6, pure OBC, and the PA6/OBC and PA6/OBC/POE-g-MAH blends with different contents of POE-g-MAH as a function of frequency. It was observed that PA6 exhibited a Newtonian behavior in the lower frequency region followed by a shear-thinning behavior in the higher frequency region, while OBC showed a typical shear-thinning behavior over the entire studied range. This may be due to the much more rigid structure of PA6 compared with OBC. It was also noted that the addition of OBC enhanced the shear-thinning behavior of pure PA6 and caused the Newtonian plateau to shift toward lower frequencies. This behavior was more visible in the PA6/OBC/POE-g-MAH blends, which indicated that compatibilizer POE-g-MAH with flexible chains contributed a lot to the observed shear-thinning behavior. In addition, the complex viscosity of pure PA6 increased appreciably with the addition of OBC. This was probably due to the inherent larger melt-viscosity of OBC and more entanglement points formed in the melt caused by the addition of OBC, resulting in the larger viscosity of the uncompatibilized PA6/OBC blend.

With the incorporation of POE-g-MAH, it can be seen that the complex viscosity of PA6/OBC/POE-g-MAH blends increased gradually with increasing POE-g-MAH contents, especially in the low frequency range. To get a straightforward comparison, the complex viscosity values of pure PA6, pure OBC, PA6/OBC and PA6/OBC/POE-g-MAH blends with different amounts of POE-g-MAH at the frequency of 10^−1^ rad/s were summarized in Table 2. As can be seen from this table, the complex viscosity of all samples at low frequency increased in the order of: PA6 < PA6/OBC < PA6/OBC/1 < PA6/OBC/3 < PA6/OBC/5 < PA6/OBC/7 < OBC. The results of increase in the complex viscosity of the compatibilized PA6/OBC blends were attributed to the chemical reactions of the maleic anhydride groups of POE-g-MAH with the amine groups of PA6 (Figure 3). As shown in Figure 3, the formation of the POE-g-PA6 copolymer at the interface of the blends caused the enhancement of the macromolecular entanglements and inhibited the flow of the melt, resulting in an increased complex viscosity of the blends. Similar behavior was observed in other compatibilized PA6 based blends [15]. In addition, at a higher frequency, the disentanglement of the molecular entanglements decreased the complex viscosity of the blends.

**Figure 7:**
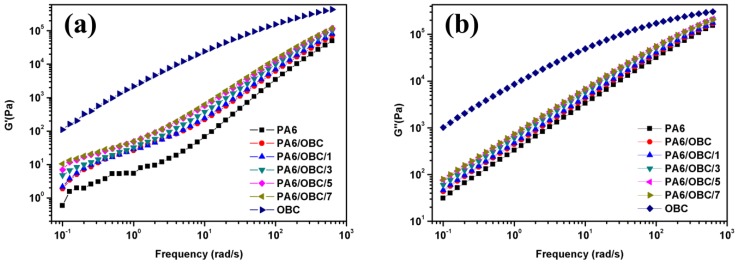
(**a**) Storage modulus (G’) and (**b**) loss modulus (G”) of pure PA6, pure OBC, and the PA6/OBC and PA6/OBC/POE-g-MAH blends at various POE-g-MAH contents.

Figure 7 illustrates the storage modulus (G’) and the loss modulus (G”) of pure PA6, pure OBC, and the PA6/OBC and PA6/OBC/POE-g-MAH blends with different contents of POE-g-MAH. As depicted in Figure 7a, the G’ of the samples in the entire frequency interval exhibited a similar trend to that of the η*. It was clear that the addition of OBC increased the G’ of pure PA6, and the addition of POE-g-MAH further increased the G’ of the blends. For instance, as shown in Table 2, at the frequency of 10^−1^ rad/s, the values of the storage modulus for PA6/OBC/POE-g-MAH blends increased from 1.9 to 10.5 Pa with increasing POE-g-MAH ratio from 0 to 7 wt%, which were all much higher than that of pure PA6 (0.6 Pa).

It has been reported that the storage modulus relates to the elastic behavior of the materials and represents the amount of stored energy [42]. Therefore, for the uncompatibilized PA6/OBC blend, the flexible macromolecules of OBC were able to store further elastic energy, and were thus responsible for the increased storage modulus of the PA6. For the compatibilized PA6/OBC blends, the increase in G’ might have resulted from the compatibilizing efficiency of POE-g-MAH. After the addition of POE-g-MAH to the PA6/OBC blend, the PA6 matrix had a stronger interaction with OBC particles through the interface by reducing the interfacial tension.

The higher storage modulus values of the compatibilized PA6/OBC blends point out that the addition of the compatibilizer to the blends generated larger elasticities, indicating improved compatibility of the PA6/OBC blends. Figure 7b shows a trend of G” similar to that of G’. In addition, it was observed that G” was higher than G’ over the entire frequency range of testing indicating thus that all samples exhibited a liquid-like behavior, and that the compatibilized blends are viscoelastic materials [43]. Arsad et al. [44] also reported liquid-like behavior in the study of compatibilized PA6/ABS blends. This study indicated that this behavior was due to the fact that all the samples were predominately made of PA6 phase and had good processability. It is interesting to note that the result above is in correspondence with the result derived from the morphological analyses above.

### 3.5. Thermal Behavior of The Materials

**Figure 8:**
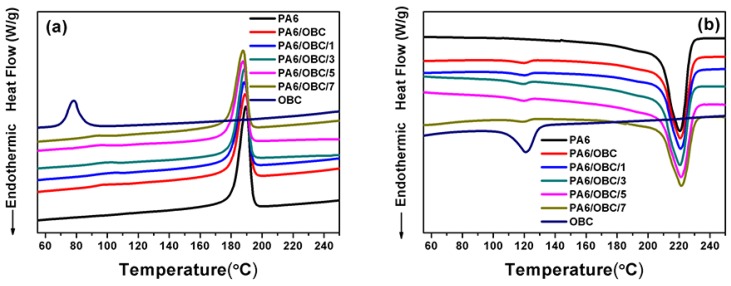
The differential scanning calorimetry (DSC) plots of (**a**) the cooling curves and (**b**) the second heating curves of pure PA6, pure OBC, and the PA6/OBC and PA6/OBC/POE-g-MAH blends at various POE-g-MAH contents.

The DSC cooling curves and the second heating curves of pure PA6, pure OBC, and the PA6/OBC and PA6/OBC/POE-g-MAH blends with different contents of POE-g-MAH are given in Figure 8. The results of T_m_, ΔH_m_, T_c_, and X_c_ are summarized in Table 3. The data revealed that the addition of OBC and POE-g-MAH had little effect on the T_m_ of PA6 in the uncompatibilized and compatibilized blends. However, the T_c_ of PA6 in all the mixtures was only influenced by the addition of the POE-g-MAH compatibilizer. Indeed, the T_c_ of PA6 gradually shifted to lower temperatures with the addition of POE-g-MAH to PA6/OBC blend. When the dosage of POE-g-MAH increased from 0 to 7 wt%, the T_c_ of PA6 in the compatibilized blends decreased from 189.05 to 187.67 °C, which suggested that the addition of POE-g-MAH had a negative effect on the crystallization of PA6. The reason for reducing the crystallization temperature of PA6 might be due to the formation of the POE-g-PA6 copolymer resulting from the reaction between PA6 and POE-g-MAH, which hindered the crystallization of PA6.

In addition, from Figure 8b, it can be seen that, compared with the pure PA6, the PA6/OBC and the PA6/OBC/POE-g-MAH blends exhibited two melting peaks in the heating process. Here, the major endothermic peak located at around 220 °C was attributed to the melting of PA6, and the small peak located at around 120 °C was attributed to the melting of OBC [45]. This was because the OBC consisted of crystallizable ethylene-octene blocks [35]. In addition, the X_c_ of the pure PA6, the PA6/OBC and the PA6/OBC/POE-g-MAH blends with a lower dosages (1–5 wt%) of POE-g-MAH obtained from the DSC heating thermograms was around 31%, pointing out that the crystallinity of PA6 was not significantly affected by the OBC or POE-g-MAH at the low contents. However, at a 7 wt% addition of POE-g-MAH, the X_c_ of PA6 decreased to 26.92%, 4.74% lower than that of pure PA6. When the addition of POE-g-MAH exceeded a certain amount, the crystallization ability of PA6 decreased because of the chemical interactions between the POE-g-MAH and PA6 matrix, which limited the molecular chain mobility of the PA6 that is necessary for crystallization, resulting thus in a decrease in PA6 crystallinity.

### 3.6. Mechanical Properties

**Figure 9:**
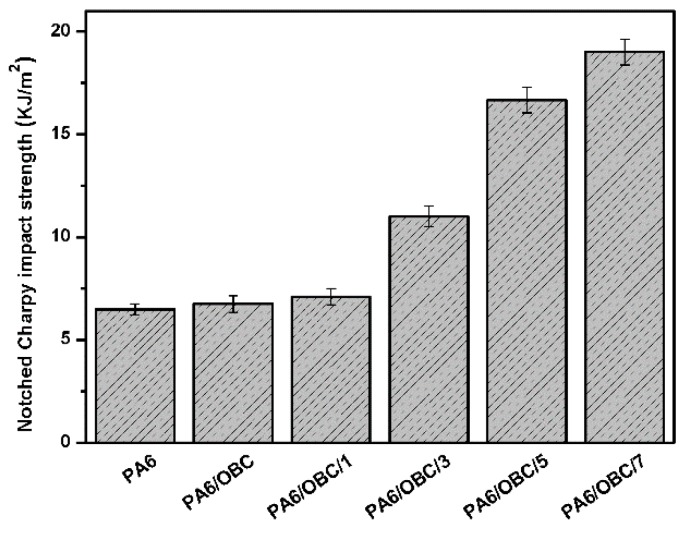
Notched Charpy impact strength of pure PA6, and the PA6/OBC and PA6/OBC/POE-g-MAH blends at various POE-g-MAH contents.

Values of the notched Charpy impact strength (IS) of pure PA6, PA6/OBC and the PA6/OBC/POE-g-MAH blends with various contents of POE-g-MAH are shown in Figure 9. As can be seen, very limited increments in IS were obtained in the case of uncompatibilized PA6/OBC blend as compared to that of pure PA6, which might be ascribed to the poor compatibility between the PA6 matrix and OBC phases as demonstrated by the SEM micrographs in Figure 5a. However, when POE-g-MAH was added to the PA6/OBC blend, the IS of the compatibilized blends increased significantly with increasing POE-g-MAH contents. In particular, the PA6/OBC/7 sample exhibited the highest IS (19 kJ/m^2^), which was about 194% higher than that of pure PA6 under our experimental conditions.

This successful toughening improvement of PA6 could be due to the chemical reaction of the maleic anhydride groups of POE-g-MAH with the amine groups of PA6 to form the POE-g-PA6 copolymer, which could act as bridges between PA6 matrix and OBC phase to enhance the compatibility of the PA6/OBC blends. In addition, considering the corresponding SEM images, it is believed that the increase of IS might have resulted from the inhibition of the coalescence of the dispersed phase and the promoted dispersion of OBC particles in the PA6 matrix, as well as the improved interfacial adhesion and molecular chain entanglements between the two phases, which allowed for efficient stress transfer between the phases and caused a large yield deformation, which dissipated large amounts of energy responsible for the improved IS of the blends [46,47]. The toughening effect of maleic anhydride functionalized elastomer particles has also been reported by other researchers [48,49]. The obtained results indicate that the compatibility of the PA6/OBC blend was greatly improved by the incorporation of POE-g-MAH.

**Figure 10:**
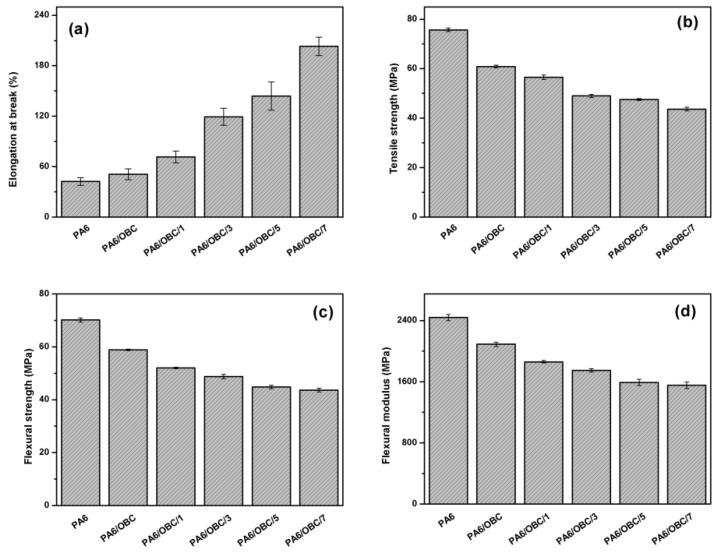
(**a**) The elongation at the break, (**b**) the tensile strength, (**c**) the flexural strength, and (**d**) the flexural modulus of pure PA6, and the PA6/OBC and PA6/OBC/POE-g-MAH blends at various POE-g-MAH contents.

Figure 10 depicts the tensile and flexural properties of pure PA6, and the PA6/OBC and PA6/OBC/POE-g-MAH blends with different contents of POE-g-MAH. As expected, the incorporation of OBC increased the elongation at break of pure PA6. This might be attributed to the elastomeric nature of OBC. In addition, the elongation at the break of the compatibilized blends gradually increased with the increase of POE-g-MAH contents, and reached the maximum value at the concentration of 7 wt% POE-g-MAH, which was about 3.8 times higher than that of pure PA6. This could be ascribed to the improved interfacial adhesion between the PA6 matrix and OBC phase resulting from the formation of the POE-g-PA6 copolymer at the interface, which increased the plastic deformation or ductility of the blend. However, owing to the elastomeric nature of OBC and POE-g-MAH, the addition of OBC and POE-g-MAH decreased the tensile strength, flexural strength, and flexural modulus of the PA6.

## 4. Conclusions

In this work, olefin block copolymer (OBC) was used as an impact modifier for PA6, along with a commercialized POE-g-MAH as a reactive compatibilizer for the PA6/OBC blend. Blends of PA6/OBC (PA6/OBC = 90/10 *w*/*w*) were prepared by melt blending in the presence of 0–7 wt% POE-g-MAH contents. The effects of POE-g-MAH on the morphology, rheological behavior, thermal behavior, and mechanical properties of the blends were investigated. The morphological analysis showed that pure PA6 and OBC were immiscible with sea-island type morphologies. SEM investigation indicated that the addition of 7 wt% POE-g-MAH to the PA6/OBC blend enhanced the dispersion of the OBC particles in the PA6 matrix with a blurred phase interface, suggesting a better interfacial compatibility between the pure PA6 and the OBC, which was consistent with the results of the rheological analysis.

The rheological test showed that the addition of POE-g-MAH increased the melt viscosity, storage modulus (G’), and loss modulus (G”) of the compatibilized blends at low frequencies, mainly due to the reaction between the maleic anhydride groups of POE-g-MAH and the amine groups of PA6, which was confirmed by Fourier transform infrared (FTIR) spectroscopy analysis and the Molau test. Moreover, DSC analysis demonstrated that the addition of OBC had little effect on the crystallization behavior of pure PA6, while the incorporation of POE-g-MAH at high concentrations (7 wt%) in PA6/OBC blend hindered the crystallization of PA6. In addition, the notched Charpy impact strength and the elongation at break of the PA6/OBC blend increased with increasing POE-g-MAH compatibilizer content, and a maximum impact strength of 19 kJ/m^2^ was reached for the blend PA6/OBC/7, which was approximately 194% higher than that of pure PA6 under our experimental conditions. However, owing to the elastomeric nature of OBC and POE-g-MAH, the addition of OBC and POE-g-MAH decreased the tensile strength, flexural strength, and flexural modulus of the PA6.

## Figures and Tables

**Table 1:** The compositions of the studied blends.

Sample Code	Polyamide 6 (PA6) (wt%)	Olefin Block Copolymer (OBC) (wt%)	Maleic Anhydride-Grafted Polyethylene-Octene Copolymer (POE-g-MAH) (wt%)
Pure PA6	100	0	0
PA6/OBC	90	10	0
PA6/OBC/1	90	10	1
PA6/OBC/3	90	10	3
PA6/OBC/5	90	10	5
PA6/OBC/7	90	10	7

**Table 2:** The complex viscosity (η*), storage modulus (G’), and loss modulus (G”) of pure PA6, pure OBC, and the PA6/OBC and PA6/OBC/POE-g-MAH blends at various POE-g-MAH contents and a frequency of 10^−1^ rad/s.

Sample	Complex Viscosity (η*) (Pa·s)	Storage Modulus (G’) (Pa)	Loss Modulus (G”) (Pa)
Pure PA6	330.0	0.6	31.1
Pure OBC	10472.9	110.6	1018.3
PA6/OBC	461.0	1.9	43.3
PA6/OBC/1	485.5	2.2	46.6
PA6/OBC/3	602.6	4.7	60.0
PA6/OBC/5	758.4	6.9	75.6
PA6/OBC/7	806.6	10.5	80.0

**Table 3:** The DSC results of pure PA6, pure OBC, and the PA6/OBC and PA6/OBC/POE-g-MAH blends at various POE-g-MAH contents.

Sample	T_m_ (°C)	T_c_ (°C)	∆H_m_ (J/g)	X_c_ (%)
Pure PA6	220.78	189.55	72.82	31.66
Pure OBC	121.06	78.34	18.0	6.21
PA6/OBC	220.86	189.05	65.85	31.81
PA6/OBC/1	220.87	188.77	63.15	30.81
PA6/OBC/3	220.67	188.62	63.98	31.84
PA6/OBC/5	221.17	187.78	61.57	31.23
PA6/OBC/7	221.46	187.67	52.08	26.92
